# Tacrolimus-Induced Diffuse Coronary Artery Spasm

**DOI:** 10.7759/cureus.25748

**Published:** 2022-06-08

**Authors:** Abadil Samer, Fahad Almehmadi, Ahmed Krimly, Abdullah Alrajhi

**Affiliations:** 1 Medicine, King Saud Bin Abdulaziz University for Health Sciences, Jeddah, SAU; 2 Cardiology, King Abdulaziz Medical City, Jeddah, SAU; 3 Cardiology, King Faisal Cardiac Center, King Abdulaziz Medical City, Jeddah, SAU

**Keywords:** coronary artery spasm, renal failure, solid organ transplant, tacrolimus, immunosuppressant

## Abstract

Prinzmetal angina, also known as vasospastic angina, is defined as an intermittent focal or diffuse coronary artery narrowing, which is often associated with transient ST-segment elevation on an electrocardiogram. Also, it could be associated with an atherosclerotic lesion at the site of the spasm. Vasospastic angina might be induced by medications, most commonly with cocaine and other examples which include catecholamines such as epinephrine, norepinephrine, isoproterenol, dopamine, and dobutamine. Parasympathomimetic agents include acetylcholine, methacholine, and pilocarpine. It is rarely caused by tacrolimus. The clinical evaluation includes an electrocardiogram and echocardiogram. The confirmed diagnosis is done by coronary angiography. Cardiac catheterization is indicated in such cases to rule out coronary artery disease.

## Introduction

Tacrolimus is a cornerstone immunosuppressant for most solid organ transplant recipients [1]. It functions through calcineurin inhibition that subsequently reduces T-lymphocyte proliferation. Vasoconstrictive properties of tacrolimus contribute to its commonly encountered nephrotoxicity. Cardiovascular toxicity manifests in the form of hypertension, but diffuse multi-vessel coronary spasms are rarely encountered [2]. Here, we report a case of presumed tacrolimus-induced multi-vessel coronary artery spasm.

## Case presentation

**Figure 1:**
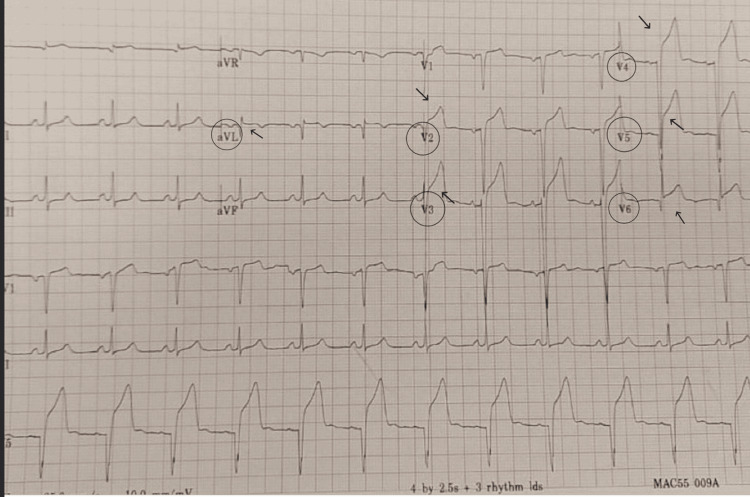
Electrocardiogram (ECG) showing ST-elevation in V2-6 and aVL

A 35-year-old male with a known history of hypertension, end-stage renal disease (ESRD), and a non-smoker male, received a living donor renal transplant that was done in another country. Subsequently, his graft progressively failed, and the patient ended up back on dialysis for the last five years. To prevent graft-antibody-mediated rejection, the patient was kept on oral tacrolimus and prednisolone. The patient presented to the emergency room with severe respiratory distress and cardiogenic shock. On initial evaluation, the patient’s vitals showed a temperature of 97.2°F, blood pressure of 152/87 mmHg, heart rate of 107 beats per minute, respiratory rate of 30 breaths per minute, and oxygen saturation of 98% on room air. Lab work was done at that time without significant results. His presenting electrocardiogram (ECG) showed diffuse ST-segment elevation along with significant elevation of high sensitivity troponin (hs-Trop) at >180,000 units (Figure 1). With prolonged vasospasm, elevated high sensitivity troponin might be seen. Catheterization lab was immediately activated for an emergency. His tacrolimus level at that time was within acceptable at 5.10 ng\mL. The normal tacrolimus serum range is (3-7 ng/mL).

**Figure 2:**
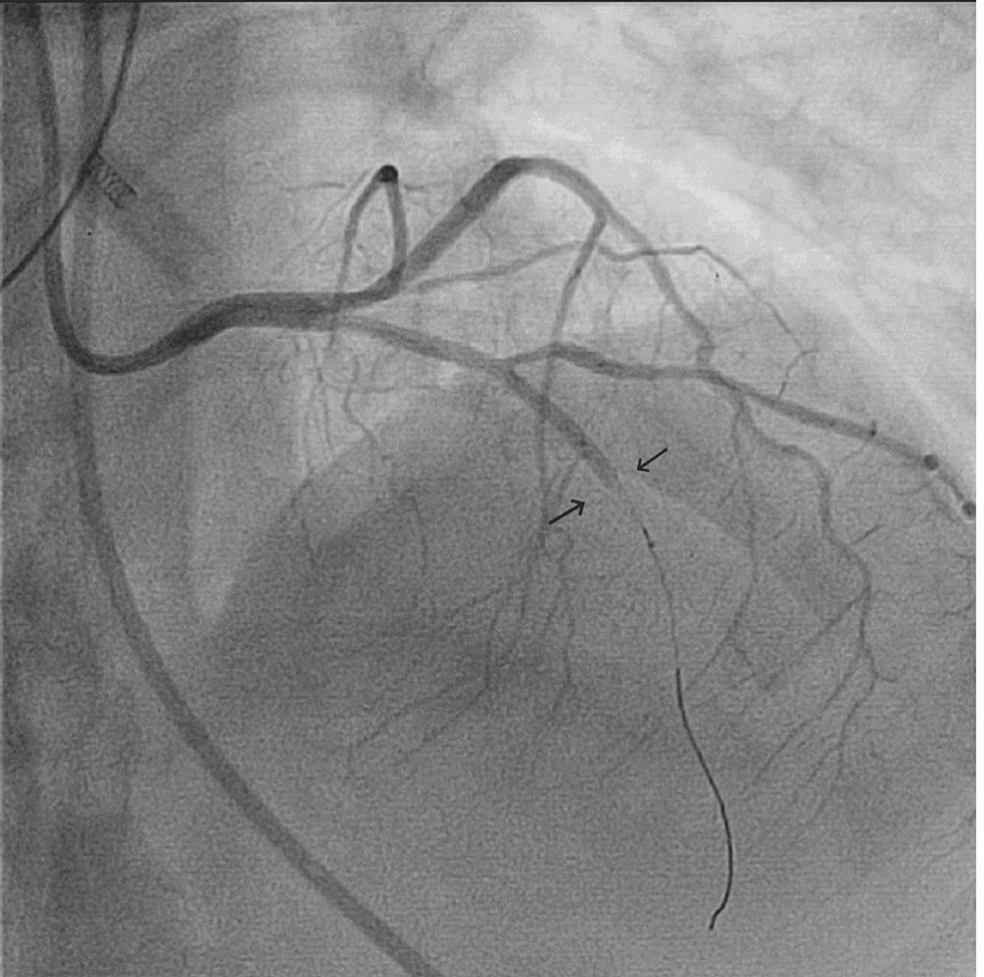
Severe left anterior descending (LAD) artery spasm

The coronary angiogram showed severe left anterior descending (LAD) artery spasm, osteal-mid 99.9% diffuse spasm followed by a normal artery with no clear coronary artery disease (Figure 2). Suddenly, the LAD artery showed no flow distally and the patient had a severe drop in blood pressure and loss of pulse. Cardiopulmonary resuscitation (CPR) was immediately started for six minutes, and intra-coronary nitroglycerin 200 μg was injected without significant improvement in coronary flow. The patient continued to be hypotensive, and the decision was to place an intra-aortic balloon pump (IABP) for support.

Intravascular ultrasound was done and showed no evidence of underlying coronary disease, dissection, or thrombus. The distal-mid-proximal left anterior descending artery shows no significant coronary artery disease (Video 1). Attempted percutaneous coronary intervention (PCI) failed to restore coronary flow. The decision was to opt for a conservative strategy and redo the coronary angiogram in 24-48 hours. Consulting critical care and transplant nephrology, the decision was to hole tacrolimus, meanwhile, as the culprit for the patient’s presentation. The patient was transferred to the critical care unit. Laboratory analysis was done and showed glucose levels of 10 mg/dL (reference range: 0-15 mg/dL). Other significant results were neutrophils of 81.7% (reference range: 34-67.9%), potassium of 3.3 mEq/L (reference range: 3.5-5.3 mEq/L), creatinine of 1.5 mg/dL (reference range: 0.6-1.2 mg/dL), and magnesium of 0.7 mEq/L (reference range: 1.3-2.1 mEq/L). The calcium level was 1.25 mg/dL (reference range: 8.6-10.3 mg/dL) and phosphorus was 2.11 mg/dL (reference range: 2.8-4.5 mg/dL). The patient's blood glucose was 88 mg/dL (70-99 mg/dL). Coagulation studies and liver function tests were within the normal limit.

**Video 1:** Intravascular ultrasound was done and showed no evidence of underlying coronary disease

During the admission, (IABP) protocol was followed. The patient was treated daily with a magnesium oxide of 400 mg orally. Clopidogrel 75 mg, prednisolone 5 mg, and atorvastatin 40 mg were given orally in the morning. heparin 5000 U/ml injection was given every 12 hours along with acetaminophen injection of 750 mg every eight hours. Clonidine 0.1 mg was given orally to keep him hemodynamically stable.

**Figure 3:**
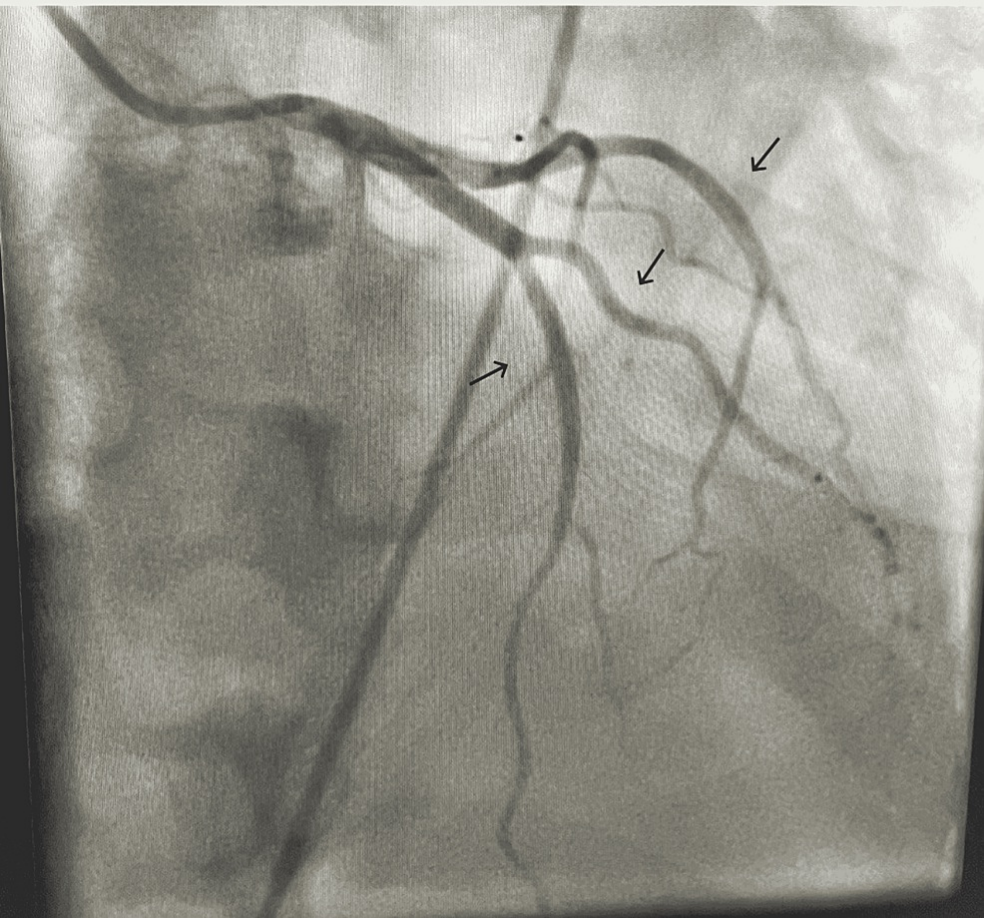
Coronary angiogram two days later showed complete resolution of the coronary spasm

A relook coronary angiogram two days later showed complete resolution of the coronary spasm (Figure 3). An echocardiogram was done four days later and showed left ventricular hypertrophy. Also, apical echoes consistent with trabeculae were noted (Video 2). Left ventricular ejection fraction dramatically improved from 20% with multi-territory wall motion abnormalities to 36%.

**Video 2:** An echocardiogram was done four days later and showed left ventricular hypertrophy

## Discussion

The coronary spasm can be induced by medication, most common with cocaine. Other examples include catecholamines (epinephrine, norepinephrine, isoproterenol, dopamine, dobutamine), parasympathomimetic agents (acetylcholine, methacholine, pilocarpine), and rarely tacrolimus [3].

The coronary spasm induced by tacrolimus is usually seen in patients with a high concentration level of the drug. The mechanisms underlying vasoconstriction induced by tacrolimus have different theories. First, tacrolimus binds FK-binding protein (FKBP) and forms a complex which then inhibits calcineurin, a Ca^2+^-dependent phosphatase. Thus, it increases intracellular calcium concentration, a known spam trigger [4]. Second, calcium channel blockers (CCB) are often used to control hypertension in kidney transplant patients [5], and both drugs, tacrolimus and CCB, are metabolized by the CYP3A system, which decreases the clearance of tacrolimus and increases the drug blood level to reach a toxic level [6].

Nitrates sublingually or intravenously should be used to decrease the risk of cardiac ischemic attacks, and they remain the mainstay of treatment to relieve the attacks of spasms [7]. The role of cardiac catheterization in such cases is to rule out coronary artery disease as the underlying cause. For patients with no evidence of coronary artery disease, further investigations should be done to exclude any other causes and to prevent episodes from reoccurring [8].

Coronary spams are treated acutely by CCB. They relieve vasoconstriction and promote vasodilation in coronary circulation. Complete or near reduction of coronary artery spasm events is observed with non-dihydropyridine CCBs more than dihydropyridine CCBs. Although coronary spasms might lead to fatal arrhythmias, the implantable cardioverter-defibrillator (ICD) indication is not clear. It depends on the case. There is no clear indication of ICD as primary prevention in coronary artery spasms [9].

The nifedipine was held in our case to prevent any possible vasoconstriction. We can prevent this side effect by doing a dose adjustment or switching to a mammalian target of rapamycin (mTOR) inhibitor, i.e., sirolimus or everolimus, to prevent any further cardiovascular adverse events [10].

## Conclusions

Physicians, especially in emergency department settings at transplant centers, should be aware of this rare but serious cardiac complication of commonly used immunosuppressants among transplant patients. Furthermore, it is important to highlight that the management of coronary spasms in the setting of tacrolimus is different. 
